# Displacement and disease: The Shan exodus and infectious disease implications for Thailand

**DOI:** 10.1186/1752-1505-2-4

**Published:** 2008-03-14

**Authors:** Voravit Suwanvanichkij

**Affiliations:** 1 Center for Public Health and Human Rights, Johns Hopkins Bloomberg School of Public Health, Baltimore, Maryland, USA

## Abstract

Decades of neglect and abuses by the Burmese government have decimated the health of the peoples of Burma, particularly along her eastern frontiers, overwhelmingly populated by ethnic minorities such as the Shan. Vast areas of traditional Shan homelands have been systematically depopulated by the Burmese military regime as part of its counter-insurgency policy, which also employs widespread abuses of civilians by Burmese soldiers, including rape, torture, and extrajudicial executions. These abuses, coupled with Burmese government economic mismanagement which has further entrenched already pervasive poverty in rural Burma, have spawned a humanitarian catastrophe, forcing hundreds of thousands of ethnic Shan villagers to flee their homes for Thailand. In Thailand, they are denied refugee status and its legal protections, living at constant risk for arrest and deportation. Classified as "economic migrants," many are forced to work in exploitative conditions, including in the Thai sex industry, and Shan migrants often lack access to basic health services in Thailand. Available health data on Shan migrants in Thailand already indicates that this population bears a disproportionately high burden of infectious diseases, particularly HIV, tuberculosis, lymphatic filariasis, and some vaccine-preventable illnesses, undermining progress made by Thailand's public health system in controlling such entities. The ongoing failure to address the root political causes of migration and poor health in eastern Burma, coupled with the many barriers to accessing health programs in Thailand by undocumented migrants, particularly the Shan, virtually guarantees Thailand's inability to sustainably control many infectious disease entities, especially along her borders with Burma.

## 

As I left the hospital, Sai Harn struggled to prop himself up from the bed, his emaciated arms upraised, his palms pressed together in a traditional goodbye. I never saw him again. Sai Harn, an ethnic Shan from southern Shan State, Burma, fled his home for Chiang Mai about a decade ago. He last worked in agriculture, finally stopping after losing weight and becoming too tired. He was diagnosed with AIDS and tuberculosis. As a migrant worker, he was ineligible for the Thai government's anti-retroviral treatment programs, and died soon thereafter. His funeral, at a local Shan temple, was attended by only a handful of people, almost all staff of a migrant safe-house where he spent his final days. His worldly possessions, including his life-savings of about 500 baht, were given away. In death, he was as invisible as he was in life, yet another tragedy in the catastrophe of Shan State.

Burma, particularly the frontiers of the country, is ethnically diverse, and perhaps a third of her peoples are non-Burman (the last census detailing ethnic makeup was done in 1931). The country has fourteen administrative divisions, of which seven are ethnic states, named after the largest ethnic group inhabiting it [1]. Shan State, bordering Thailand, Laos, and China, is the largest, covering 20% of the country's land mass. Much of it has been ravaged by five decades of continuous, low-intensity civil conflict as armed groups vied for autonomy, ideology, and business interests, including the narcotics trade. Starting in 1996, the Burmese military or *Tatmadaw*, in an attempt to expand central control, intensified its counter-insurgency strategy, the Four Cuts Policy, in central and southern Shan State [2]. The cornerstone of this policy was the forced relocation of civilians from contested areas to "relocation centers" more firmly under Rangoon's control, and destroying rice fields and food storage facilities [2,3]. Between 1996–1998 alone, over 1,400 villages in a 7,000 square mile area of central and southern Shan State, affecting perhaps 300,000 villagers, were systematically depopulated by the *Tatmadaw*[2,4]. Forced relocation was accompanied by widespread abuses of civilians by the Burmese army, including rape, confiscation of land and property (including arbitrary taxation), torture, and extrajudicial executions [2,4,5]. Rape and sexual violence by Burmese soldiers against ethnic women and girls has been particularly well-documented, including against Shan women, used as a weapon of warfare to intimidate civilians [5,6]. These abuses, coupled with ongoing conflict and failed Burmese economic policies that have drastically reduced agricultural production, worsening poverty and food insecurity, have driven perhaps 400,000 villagers from their homes in Shan State, forcing them to live as internally displaced persons (IDPs) or as migrants in Thailand [2,3,7,8]. More recently, large infrastructure projects such as dams on the Salween River, joint ventures between Thailand and the Burmese government, have resulted in increased Burmese militarization of vast areas of Shan and Karen States, accompanied by widespread abuses of civilians, displacing thousands more villagers [9,10] (Figure 1).

**Figure 1:**
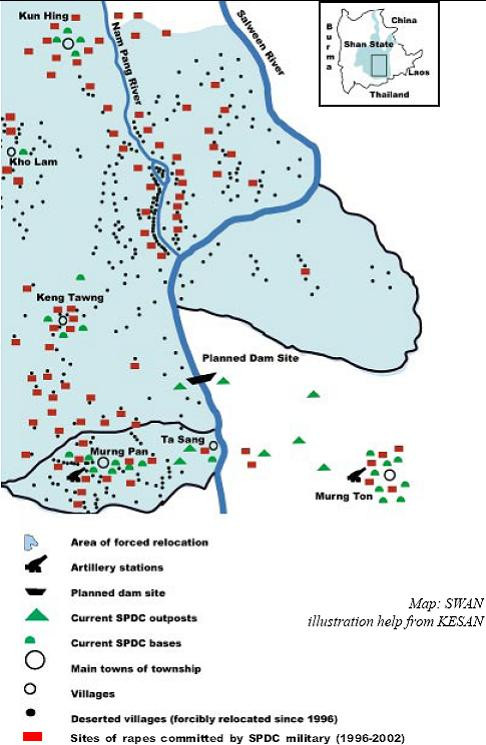
Increased militarization and sexual violence around a planned Salween dam site in Shan State, 1996–2002.

IDPs, living in fragmented communities in the jungles, face multiple dangers. *Tatmadaw *patrols often rape, torture, or kill civilians found outside permitted zones [2-4]. Forced labor or confiscation/destruction of food by Burmese troops is also common [11]. Health services are almost non-existent, and health indicators such as maternal, infant, and child mortality rates in IDP communities more closely resemble those of Angola, Sierra Leone, and Rwanda, higher than Burma's official figures, already amongst the worst in the region [11]. Most deaths are from infectious diseases, particularly malaria [11,12].

Those who have crossed the border into Thailand face other challenges. Although 140,000 who have fled Burma have been recognized as refugees, living in nine official camps in Thailand, most of these are ethnic Karen and Karenni; there are no official refugee camps for the Shan, leaving them bereft of official channels of humanitarian aid [13-15]. Most are instead classified as "economic migrants," forced to work, usually in agriculture, construction, domestic work, and the vast Thai sex industry [13,16,17]. Work conditions are often exploitative, entailing long hours for pay well below Thailand's legal minimum wage and, without official documentation, migrants constantly risk arrest and deportation. [18,19] Indeed, they tolerate abusive work conditions as these are deemed less threatening than deportation back to the conditions from which they fled [13,17]. Every year, many are injured, sickened, or lose their lives from workplace exposures (particularly pesticides), occupational accidents, and physical (including sexual) assault, the majority of which go unreported [15,18-20]. In the 1990s, demand for cheap labor in Thailand prompted implementation of a guest worker program, which provides access to Thailand's universal health plan. However, the many restrictions and complicated measures registration entails, in addition to misunderstanding, language barriers, discrimination, registration costs and other expenses bar most migrants from Burma, particularly Shans, from being legally documented [21,22]. These same barriers to legal status also bar many from accessing healthcare in Thailand, even for those who have legally registered [23].

Given the situation facing most Shan migrants, health data on this population is scant, but what data is available highlights their precarious situation. Pregnant Shan women often lack antenatal care, and easily preventable conditions such as malnutrition and neonatal tetanus are common [21,22]. Shan children often have never had or frequently miss childhood immunizations, a gap that threatens control of vaccine-preventable illnesses in Thailand, particularly polio [22,24,25]. Migrants from Burma, including the Shan, already bear a disproportionate burden of infectious disease morbidity and mortality. Tuberculosis is the most common infectious disease diagnosed on health screening of guest worker registrants, and the surge in cases, especially in Shans and other ethnic minorities living along the borders of northern Thailand, is straining the capacity of local TB control programs to isolate, treat, and follow-up patients [26,27]. Today, TB cure and treatment completion rates in migrants from Burma are consistently lower than in Thais; in one analysis in Chiang Rai Province in northern Thailand, home to thousands of Shans, only a quarter of non-Thais with TB were cured [28,29]. This problem is compounded by the high rates of HIV infection in Shan State and Shan migrants living in northern Thailand; HIV prevalence rates in this population were amongst the highest of all ethnic minorities, up to 8.75% in one analysis, rates far above their northern Thai cousins, who had some of the highest HIV infection rates in Thailand [30,31]. In Chiang Mai, AIDS is now the most common disease in Shan migrants that is reported to Thai health authorities [32].

With almost no health services available at home, few Shan migrants in Thailand have ever had basic health education prior to departure, including about HIV, and misconceptions and HIV-related stigma are common [7,33]. This is true also for Shans working in the Thai sex industry, now increasingly dominated by migrants, particularly those venues with the worst working conditions [16,33-35]. Compared to their Thai counterparts, Shan commercial sex workers are less likely to consistently use condoms, and incorrect use is common [34,36]. The result has been a maturing epidemic of HIV/AIDS, accompanied by the most common opportunistic infection, tuberculosis [37,38]. For many, the gaps which create vulnerability to HIV, coupled with lack of legal status, exploitation, and lack of access to health-related services, proved to be a lethal combination, such as for Sai Harn [16,17]. These same vulnerabilities threaten re-emergence of disease entities long controlled in Thailand, such as lymphatic filariasis; in 2004, two Shan migrants in urban Chiang Mai presented for care for symptomatic lymphatic filariasis, the first time this disease entity had been seen in decades [39,40]. This finding raises concern given that most individuals infected with the main etiologic agent, *Wuchereria bancrofti*, are asymptomatic and capable vectors still exist in Thailand [39,41].

In addition to having significant public health implications, these vulnerabilities are also exacting an economic toll on Thailand as Thai public hospitals increasingly shoulder the costs of providing charity care for migrants unable to pay for their treatments, particularly since many present for care late in the course of their illnesses, when they are too ill to work, increasing the costs of care and the risk of death [35]. Today, Mae Hong Son Province, bordering Shan State and home to tens of thousands of undocumented individuals, spends over 40 million baht per year on charity care, straining healthcare budgets already stretched thin as a result of insufficient government subsidies [42,43].

The root cause of these problems is misgovernance, particularly neglect of health by the Burmese government and widespread abuses by the *Tatmadaw *against the Shan and other ethnic groups living in eastern Burma, fueling a health catastrophe and exodus to Thailand. The problem is compounded by other barriers to Shan migrants accessing vital services in Thailand, chief of which is lack of legal status, including failure to recognize many who have fled fighting and abuses as official refugees. Thailand's ongoing failure to take the Burmese regime to task for its abusive policies, coupled with Thai investment in large infrastructure projects in eastern Burma, such as hydroelectric dams on the Salween River, risk worsening an already critical situation, further driving migration and marginalization of Shans in Thailand [44]. These not only represent policy and public health failures for the Shan, the emerging picture indicates that Thailand's ongoing failure to tackle these issues comes at its own peril.

## Competing interests

The author(s) declare that they have no competing interests.

## References

[B1] International Crisis Group (ICG) Myanmar Backgrounder: Ethnic Minority Politics. http://www.crisisgroup.org.

[B2] Risser G, Kher Oum, Htun Sein (2003). Running the Gauntlet: The Impact of Internal Displacement in Southern Shan State.

[B3] Thailand Burma Border Consortium (TBBC) (2004). Internal Displacement and Vulnerability in Eastern Burma.

[B4] Shan Human Rights Foundation (SHRF) (1998). Dispossessed: A Report on Forced Relocation and Extrajudicial Killings in Shan State, Burma.

[B5] Shan Human Rights Foundation (SHRF) and Shan Women's Action Network (SWAN) (2002). License to Rape: The Burmese Military Regime's Use of Sexual Violence in the Ongoing War in Shan State.

[B6] Karen Women's Organization (KWO) (2007). State of Terror: The Ongoing Rape, Murder, Torture and Forced Labour Suffered by Women Living Under the Military Regime in Karen State.

[B7] Hyder J, Suwanvanichkij V, Tomson N, Taylor M, Beyrer C HIV Vulnerability Among Shan Migrants in Thailand [abstract]. XVI International AIDS Conference, Toronto.

[B8] Shan Relief and Development Committee (SRDC) (2006). Deserted Fields: The Destruction of Agriculture in Mong Nai Township, Shan State.

[B9] Berger S Burma is Using Dams to Drive Out Dissident Villagers. The Telegraph.

[B10] Gray D Burma Dam Plan Causes Flood of Concern.

[B11] Backpack Health Worker Team (BPHWT) (2006). http://www.geocities.com/maesothtml/bphwt/.

[B12] McGeown K Burma"s Public Service Suffering. BBC.

[B13] Caouette TM, Pack ME (2002). http://www.refugeesinternational.org/files/3074_file_burma.pdf.

[B14] SWAN (2003). Shan Refugees: Dispelling the Myths.

[B15] Kasem S Burmese Migrants: War Refugee Camps Open Their Doors. Bangkok Post.

[B16] Beyrer C (2001). Shan Women and Girls and the Sex Industry in Southeast Asia: Political Causes and Human Rights Implications. Soc Sci Med.

[B17] Leiter K, Tamm I, Beyrer C, Wit M, Iacopino V (2004). No Status: Migration, Trafficking & Exploitation of Women in Thailand.

[B18] Keenapan N Downward Mobility. Bangkok Post Outlook.

[B19] Bhumiprabhas S Migrant Workers 'Often Locked Up'. The Nation.

[B20] Sai Silp Health Conference Highlights Risks for Shan Migrants. The Irrawaddy.

[B21] Tin Tad Clinic (2006). Proposal for a Village-Based Health Care Project at Ban Mai Ton Hoong, Fang District, Chiang Mai, Thailand.

[B22] Buadaeng K Introduction to the Project and Previous Research Activities: Study of and Improving Health Communications in Foreign Migrant Labor, the Case of Shan Migrant Workers in Chiang Mai Province.

[B23] Charoensuthipan P, Treerutkuarkul A Migrants are Missing out on Medical Care: Get Few Benefits from Health Insurance Fund. Bangkok Post.

[B24] Khwankhom A Southern Provinces a Hotbed for Polio. The Nation.

[B25] Tin Tad Clinic (2007). Proposal for supporting dispensary to serve Shan Internally Displaced (IDP) Peoples opposite of Fang district, Chaing Mai Province.

[B26] Amarinsangpen S Strategic Plan to control Tuberculosis to Meet Decade-end Development Goals, BE 2558. Talk given at seminar, Update on TB Situation in Thailand and Around the World and Launch of New Project in Northern Thailand-TB Photovoice, Chiang Mai, Thailand.

[B27] Ouppinjai N TB Related Problems or Obstacles. Talk given at seminar, Update on TB Situation in Thailand and Around the World and Launch of New Project in Northern Thailand-TB Photovoice, Chiang Mai, Thailand.

[B28] Wandee P, Supawitkul S, Pinta N, Ngoentong Y, Khunkonkapan S, Kaewkampa P, Sumanapun S, Levine W, Sinsomboontong S, Mednavyn T Dual TB/HIV epidemic in northern Thailand and Myanmar Border: The vital need for bridging cross-country cooperation [abstract]. XV International AIDS Conference, Bangkok.

[B29] Sawasdiwuthipong W, Phisuthikul K, Tatip P, Ampong T, Tatip Y, Mahasakdipan P (2006). Experiences Controlling Infectious Diseases in Burmese Migrants, Amphur Mae Sot, Tak Province, 2004. Journal of Health Science.

[B30] Beyrer C, Celentano DD, Suprasert S, Sittitrai W, Nelson KE, Kongsub B, Go V, Phanupak P (1997). Widely Varying HIV Prevalence and Risk Behaviours Among the Ethnic Minority Peoples of Northern Thailand. AIDS Care.

[B31] Srithanaviboonchai K, Choi KH, van Griensven F, Hudes ES, Visutratana S, Mandel JS (2002). HIV-1 in Ethnic Shan Migrant Workers in Northern Thailand. AIDS.

[B32] WHO Thailand and Department of Disease Control, Ministry of Public Health (2005). Overview of Thai-Myanmar Border Health Situation. http://w3.whothai.org/EN/Section3/Section39.htm.

[B33] Tilney C Male Order Business. The Irrawaddy.

[B34] Guadamuz TE, Kunawararak P, Beyrer C, Pumpaisanchai J, Celentano DD (2003). Sexual Risk Behaviors and Demographic Characteristics of Male Sex Workers in Chiang Mai, Thailand, [abstract]. XV International AIDS Conference, Bangkok.

[B35] Leiter K, Suwanvanichkij V, Tamm I, Iacopino V, Beyrer C (2006). Human Rights Abuses and Vulnerability to HIV/AIDS: The Experiences of Burmese Women in Thailand. Health Hum Rights.

[B36] Guadamuz TE, Kunawararak P, Celentano DD, Pumpaisanchai J, Beyrer C Latex and Oil: Sexual Lubricant Use Among Male Sex Workers in Chiang Mai, Thailand [abstract]. XV International AIDS Conference, Bangkok.

[B37] WHO Country Office for Myanmar (2005). Health in Myanmar.

[B38] Beyrer C, Suwanvanichkij V, Mullany LC, Richards AK, Franck N, Samuels A, Lee TJ (2006). Responding to AIDS, Tuberculosis, Malaria, and Emerging Infectious Diseases in Burma: Dilemmas of Policy and Practice. PLoS Med.

[B39] Triteeraprapab S, Kanjanopas K, Suwannadabba S, Sangprakarn S, Poovorawan Y, Scott AL (2000). Transmission of the Nocturnal Periodic Strain of Wuchereria bancrofti by Culex quinquefasciatus: Establishing the Potential for Urban Filariasis in Thailand. Epidemiol Infect.

[B40] Huanok W (2005). Thailand Under Threat: How Burma's Dams Project Could Spread Disease. The Irrawaddy.

[B41] Beyrer C, Villar JC, Suwanvanichkij V, Singh S, Baral SD, Mills EJ (2007). Neglected Diseases, Civil Conflicts, and the Right to Health. Lancet.

[B42] Treerutkuarkul A Stateless Left in Healthcare Limbo. Bangkok Post.

[B43] NHSO To Cover Those Awaiting Citizenship The Nation.

[B44] Shan Sapawa Environmental Organization (Sapawa) (2006). *Warning Signs*: An Update on Plans to Dam the Salween in Burma's Shan State.

